# Stereotactic Body Radiotherapy: is less fractionation more effective in adrenal and renal malignant lesions?

**DOI:** 10.1007/s00345-024-05140-9

**Published:** 2024-07-24

**Authors:** Daniel Rivas, Alejandro de la Torre-Luque, Elena Moreno-Olmedo, Paloma Moreno, Vladimir Suárez, Ana Serradilla, Gregorio Arregui, David Álvarez, Morena Sallabanda, Antonio Lazo, María Isabel Núñez, Escarlata López

**Affiliations:** 1 Department of Radiation Oncology, GenesisCare, Málaga, Spain; 2 Department of Legal Medicine, Psychiatry and Pathology, School of Medicine, Complutense University of Madrid. CIBERSAM ISCIII, Madrid, Spain; 3 Department of Stereotactic and MR-guided Radiotherapy, Department of Oncology, Oxford University Hospitals NHS Foundation Trust, Genesiscare, Oxford, UK; 4 Independent author, Madrid, Spain; 5 Department of Radiation Oncology, Complejo Hospitalario de Jaen, Jaen, Spain; 6 Department of Physics, GenesisCare, Jerez, Granada Spain; 7 Department of Physics, GenesisCare, Jerez Spain; 8 Department of Radiation Oncology, Instituto de Radiocirugía Avanzada y Centro de Protonterapia Quironsalud, Madrid, Spain; 9 Department of Radiation Oncology, Virgen de la Victoria Clinical University Hospital, Málaga, Spain; 10 Department of Radiology and Physical Medicine, Granada University, Granada, Spain; 11 Biopathology and Regenerative Medicine Institute (IBIMER), Centre for Biomedical Research, Granada University, Granada, Spain; 12 Biosanitary Research Institute, ibs. Granada, Spain; 13 GenesisCare South Spain Chief Medical Officer, Málaga, Spain; 14 Clinical Medicine and Public Health, University of Granada, Granada, Spain

**Keywords:** Stereotactic body radiotherapy (SBRT), Stereotactic ablative radiotherapy (SABR), Adrenal malignant lesion, Renal malignant lesion, Toxicity

## Abstract

**Purpose:**

Stereotactic body radiotherapy (SBRT) has become an excellent non-invasive alternative for many patients with primary renal cell carcinoma (RCC) and adrenal malignancies (AM). The aims of this study were to analyse how tumor-, patient- and treatment-related factors may influence the outcomes and side effects of SBRT and to assess its benefits as an alternative to surgery.

**Methods:**

This retrospective, multicenter study included 25 lesions in 23 patients treated with SBRT using different devices (LINAC, CyberKnife^®^ and Tomotherapy^®^). A multivariate linear regression was used for the statistical study.

**Results:**

Local control time was higher than six months in more than 87% of patients and treatment response was complete for 73.68%. There was an overall 2-year survival of 40% and none of the deaths were secondary to renal or adrenal local progression. Patients treated with lower total radiation dose (mean [m] = 55 Gy) but less fractions with more dose per fraction (> 8.5 Gy) showed better outcome. Patients with previous chemotherapy and surgery treatments also showed higher complete response and disease-free survival (> 6 months).

**Conclusions:**

This study highlights the importance of ultra-hypofractionated regimens with higher doses per session. Thus, the referral of patients with RCC and AM to Radiotherapy and Oncology departments should be encouraged supporting the role of SBRT as a minimally invasive and outpatient treatment.

**Supplementary Information:**

The online version contains supplementary material available at 10.1007/s00345-024-05140-9.

## Background

The incidence of renal cell carcinoma (RCC) has recently increased up to 2% [1] and adrenal malignancies (AM) up to 15–35% [2]. Traditionally, surgery has been considered the standard treatment with 2-year overall survival (OS) rates of 46%, an overall complication rate of 12.5% and a mortality rate of up to 6–8% in the laparoscopic approach. However, surgery is not possible in some circumstances (bilateral tumors, solitary kidney, or pre-existing chronic renal failure). Stereotactic body radiation therapy (SBRT) has emerged as an effective alternative for these patients [3, 4].

Although different minimally invasive treatments have been used (radiofrequency ablation [RFA], cryoablation, microwave ablation, and irreversible electroporation), SBRT seems to be a promising non-invasive treatment option [5].

While RCC has traditionally been considered a radioresistant tumor, recent serial findings in RCC brain metastases have shown that it is sensitive to radiosurgery [6], so SBRT should also be effective in these primary tumors [7].

Higher Biological Equivalent Dose (BED) overcome the challenge of radioresistant histologies. Siva’s latest work in RCC (a radioresistant tumor) with a very high control [7] leads us to believe that AM (more radiosensitive) will also have similar behavior.

Despite the high frequency of AM at autopsy and the potential for many primary tumors to metastasize to the adrenal glands, SBRT remains underutilized [8, 9]. The use of radiotherapy (RT) for the treatment of AM has traditionally been palliative [8], but SBRT has changed oncologists’ view of metastatic disease [8, 10], making it an excellent option for treating RCC and AM even in patients with postoperative positive margins [11].

The anatomical proximity of the adrenal glands to the kidneys justifies the simultaneous analysis of the technical issues related to SBRT delivery at both sites [4], given that the dose constraints for Organs at risk (OAR) are the same in both cases. Although the radiosensitivity of RCC and AM is not the same due to their different histology, the high BED of SBRT usually overcomes this tumoral challenge.

The aims of this study were to analyse how tumour-, patient- and treatment-related factors may influence the outcomes and side effects of SBRT and to ssess its benefits as an alternative to surgery.

## Methods

This study is a retrospective, multicenter analysis of 25 lesions in 23 patients. Patients were treated for RCC and AM with SBRT using the same devices (Elekta^®^ Synergy LINAC, Cyberknife^®^, and Tomotherapy^®^) between 2010 (when we introduced this technique for these patients) and 2020 in five RT departments.

Each case was discussed within a SBRT Multidisciplinary Team (MDT) attended by radiation oncologists, urologists, medical oncologists, pathologist, general surgeons, radiologists, and nuclear medicine doctors. Written informed consent was obtained from all subjects.

All the patients met the following inclusion criteria:

1. *≥* 18 and *≤* 80 years old.
2. presence of solid primary tumor or postsurgery clips.
3. RCC, primary adrenal tumor or AOM.
4. AM in patients with > 1 metastatic site should also be treated.
5. life expectancy > 6 months.
6. inoperable patients or refused surgery.
7. lesions ≤ 5 cm (except 1 exceptional patient).

Although our patients were not surgical candidates or refused surgery, all of them were suitable for SBRT with ECOG = 0 and ECOG = 1 (only one).

Exclusion criteria were:

1. primary hematologic neoplasm.
2. polymetastases (> 5 lesions); or.
3. contraindications for RT such as pregnancy and connective tissue disease.

A planning computed tomographic (CT) scan was scheduled for each patient using a specific SBRT immobilization system depending on the device (supplementary Table 1), in the supine position with a slice thickness of 3 mm.

Three CT simulation scans were acquired: (1) in shallow breathing, (2) in deep inspiration, and (3) at end expiration and the images were transferred to the Treatment Planning System (TPS).

**Fig. 1:**
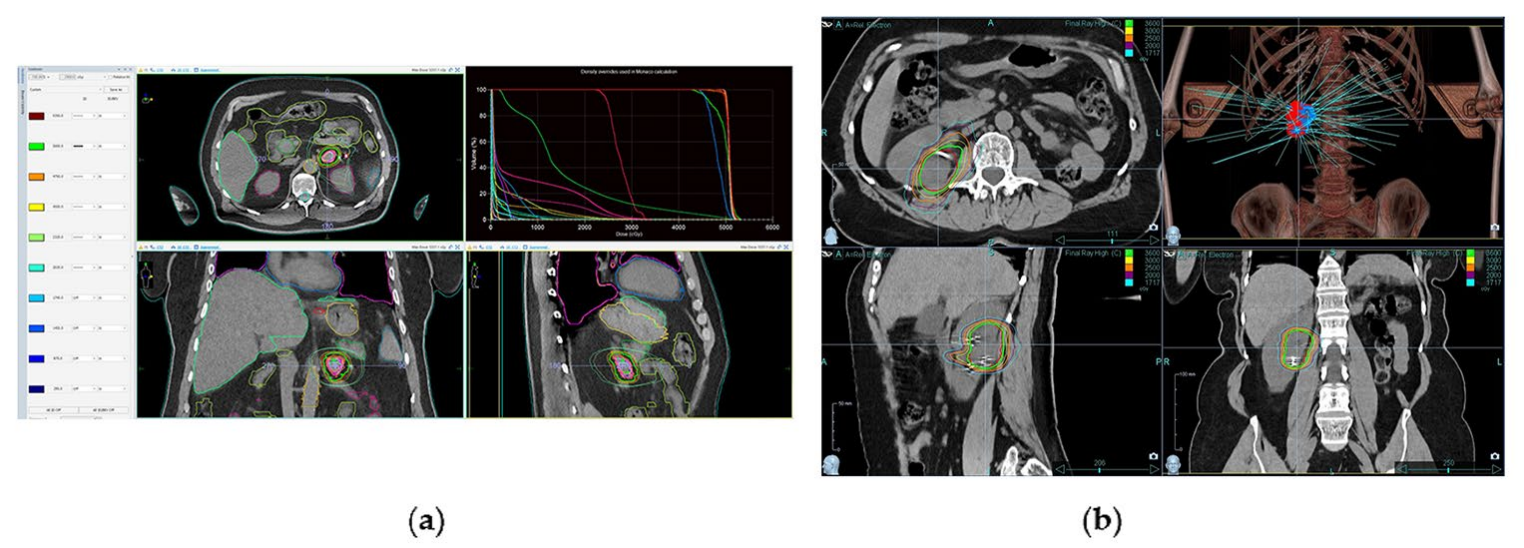
LINAC and CyberKnife^®^ dosimetry. **a**) Dosimetry in LINAC for AM and **b**) dosimetry in CyberKnife^®^ for RCC

Gross tumor volume (GTV) including all the renal or adrenal lesions were delineated. Only in one operated case with involved positive margin was considered clinical target volume (CTV). The spine, bowel, gastric, duodenum and both kidneys were contoured as organs at risk (OAR). The three GTVs from the CT scans acquired were merged into one internal target volume (ITV). A margin was added to this ITV for obtaining the planning target volume (PTV). The added margin to create the PTVs was 3–5 mm in all centers and devices. The dose constraints applied to critical organs are described in the literature [12]. We present two different plannings in the Fig. 1.

The median prescribed dose was 60 Gy (range 40–64), and the median number of fractions was 5 (range 3–8). The median volume of the lesions was 14.11 cm3 (range 4.3–111). All but three patients had a Biological Equivalent Dose with α/β = 10 (BED10) > 100 Gy. Although there is a range of alpha/beta for each individual tumor [12], we considered an α/β = 10 for all our lesions.

Dose prescription in the different machines and treatment planning systems have been: (1) LINAC and Tomotherapy^®^ following ICRU 83 criteria (20 lesions), (2) Cyberknife^®^ following the ICRU 91 criteria (5 lesions).

The Common Terminology Criteria for Adverse Events (CTCAE) 4.0 scoring system was used to evaluate side effects [13]. Toxicity results are shown in Table 1. The patient with duodenal ulcer was diagnosed by gastric endoscopy and hospitalised for conservative treatment with intravenous H2 antagonists and proton pump inhibitors by gastroenterologists.

**Table 1:** Toxicity according CTCAE v4.0

Location (No. of patients)	Side effect, grade (No. of patients)
Right adrenal gland (10)	Gastric discomfort, grade 1 (1) Diarrhea, grade 1 (1) Duodenal ulcer, grade 3 (1)
Left adrenal gland (9)	Dorsolumbar pain, grade 1 (2) Nausea, grade 1 (1)
Bilateral adrenal gland (2)	Dorsolumbar pain, grade 1 (1)
Right kidney (1)	-
Left kidney (1)	-

A follow-up visit was conducted six weeks after finishing RT and then every three months. To study local control (LC) as the main clinical endpoint, the rutinary test done along the follow up has been a CT scan. The criteria for tumor response evaluation were RECIST 1.1 [14]. Additional outcomes considered were treatment response (TR) (partial vs. complete remission), progression-free survival interval (PFS) (less than 6 months vs. 6 months or more), and OS.

A Kaplan-Meier non-parametric analysis was conducted to estimate the recurrence risk, considering our outcomes (LC, TR, PFS, and OS) and tumor-, patient-, and treatment-related factors. Regarding variables with a continuous nature (age, lesion volume, total radiation dose), the non-parametric Mann-Whitney U test was used. The same tests were conducted to determine the relationship between radiation toxicity and the clinical factors. R x64 3.0.1 software was used for all the statistical analyses.

## Results

Table 2 shows the sociodemographic and clinical characteristics of the 23 patients included in the study. The surgery addressed the same lesion location treated with SBRT. The reasons to indicate adjuvant treatment was progression of disease except for one patient with involved positive margin after surgery. There were no deaths caused by renal or adrenal local progression. The most frequent cause of death was brain metastases (*n* = 5), followed by either liver or abdominal lymph node involvement (*n* = 2 for both cases); and lung involvement or complications from abdominal surgery (*n* = 1 for both cases). In one patient, the cause of death was not known as only bone secondary disease was found. Two patients were lost to follow-up.

**Table 2:** Sociodemographic, clinical features of SBRT patients and radiotherapy parameters (*N* = 23)

Patient Characteristics	Descriptive statistics
Sex (% female)	39.13
Age (years)	60.87 (12.16)
Primary tumor site	
Lung	52.17
Esophagus	8.7
Kidney	8.7
Colon/rectum	17.39
Breast	8.7
Adrenal glands	4.35
Lesion location	
Adrenal	91.3
Renal	8.7
Previous treatment	
Surgery (%yes)	34.78
Chemotherapy (%yes)	21.74
PTV (% >50%)	60.87
BED10 (% ≥ 100 Gy)	77.86
Prescribed dose (Gy)	54.48 (9.71)
Dose per fraction (%)	
≤ 7.5 Gy	52
8–10 Gy	28
≥ 10 Gy	20
Lesion volume (cm3)	25.92 (27.40)
Follow-up time (months)	28.69 (24.61)
Local control time (% > 6 months)	87.5
Treatment response (% complete)	73.68
Free disease interval (% > 6 months)	52.94
Exitus (% died)	52.17*
Toxicity (% yes)	32

*Note* Categorical variables are expressed as percentage and continuous variables are expressed as mean and standard deviation (in brackets). RCC = renal cell carcinoma. PTV = planning target volume. PTV (% >50%) means that at least all PTVs were 50% covered following ICRU 83 criteria

BED10 = Biologically Equivalent Dose with α/β = 10. $\:BED=D\left(1+\frac{d}{\frac{\alpha\:}{\beta\:}}\right)$ Prescribed dose (Gy): Total amount of radiation therapy to be administered. * This percentage does not include those patients lost to follow-up

**Fig. 2:**
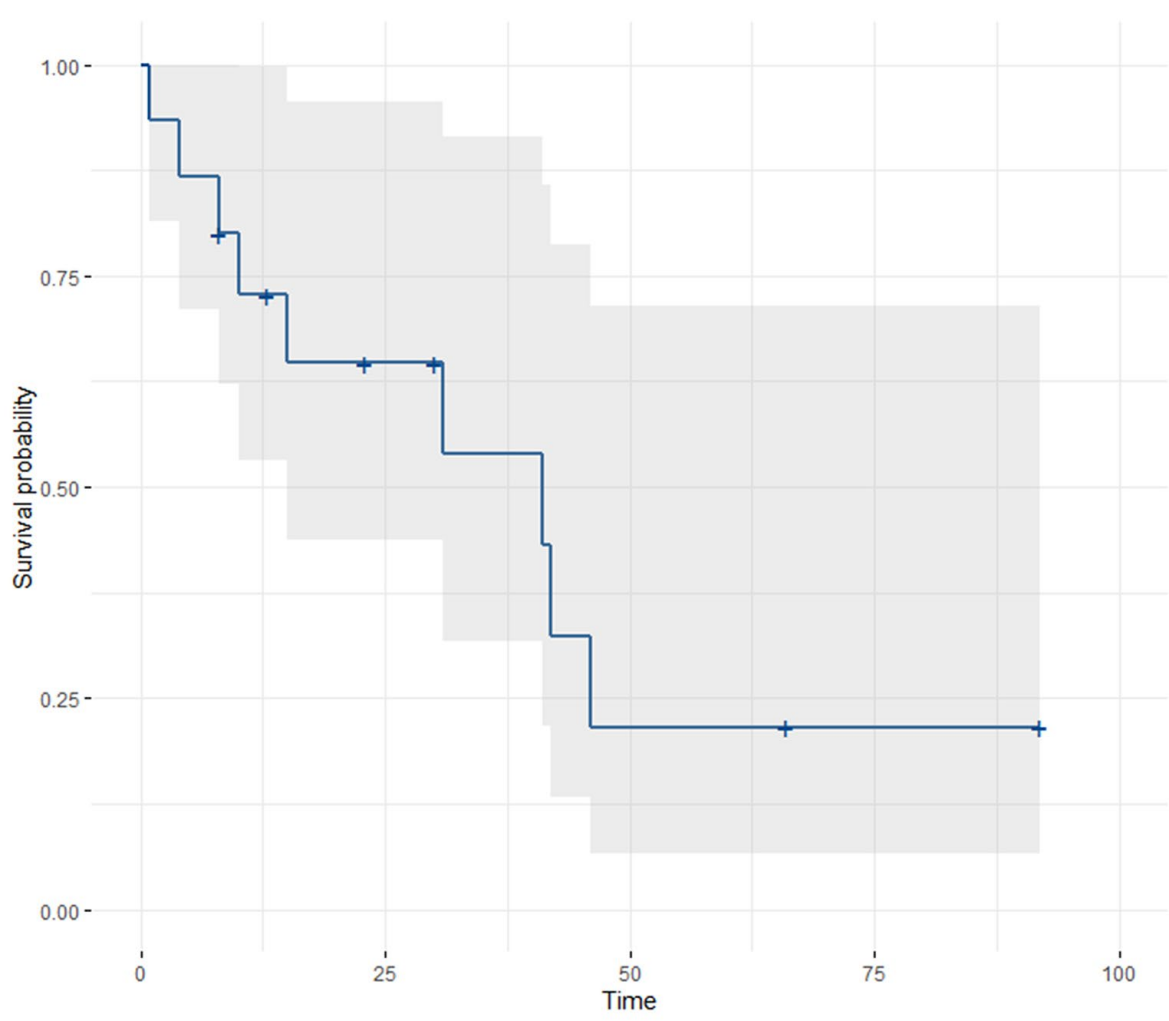
Survival model for patients treated with SBRT. Note. Shadow area depicts the 95% confidence interval of survival probability

The survival probability remained relatively high during the first year of follow-up, dropped to 0.60 at two years, and then continued to decline (Fig. 2).

Regarding mortality, four clinical factors were significantly associated with exitus: previous chemotherapy (*p* < 0.05), SBRT number of fractions (*p* < 0.05), prescribed dose (*p* < 0.01) and lesion volume (*p* < 0.01). Most of the patients (85.71%) who died during the follow-up had not undergone chemotherapy prior to SBRT. On the other hand, 37.5% of patients who underwent surgery remained alive at the study end point. Moreover, patients treated on an 8-session schedule to a dose of 7.5–8 Gy per fraction were more likely to die (80%) compared to patients treated to other SBRT modality (28.57%). Additionally, patients who died had received a higher total radiation dose (due to longer fractionation) on average (m = 59 Gy) and had lower lesion volume (m = 16.83 cm^3^) than those remained alive at the study end point (dose: m = 51.12; lesion volume: m = 45.28 cm^3^), and also lower local control (m = 55 Gy vs. m = 64 Gy) and lower free interval disease at six months (m = 52.78 vs. m = 59).

SBRT TR was associated with previous chemotherapy (*p* < 0.05) (almost 50% of patients with complete SBRT response had undergone chemotherapy) and higher BED10 (*p* < 0.05) (85% of patients with complete response, with BED10 > 100). Previous surgery (*p* < 0.05) was also associated with free interval disease outcome.

All the participants showed grade 1 toxicity according to the CTCAE v4.0 system, except one patient who showed grade 3. Relationships between toxicity indicators and clinical variables were also not significant.

## Discussion

This small study shows that patients with RCC and AM treated with SBRT have very good LC and disease-free interval at 6 months and that previous chemotherapy +/- surgery, and a high dose per fraction are better for these both clinical results. Therefore, SBRT can be an excellent option when surgery is not possible, if the patient refuses surgery or even after surgery with positive margins.

Recently, Siva et al. have published a systematic review and practice guideline supporting the practice of SBRT for RCC as a safe and effective standard treatment option [7].

Chan et al. for AM obtained an LC of 82% and 62% and OS of 66% and 42% at 1 and 2 years respectively, with a median BED of 67 Gy [15]. In our series OS was 70 and 60% at 1 and 2 years respectively, 6-month LC was 87% and 73.68% of patients achieved a complete response by imaging, with BED > 100 Gy for most patients, which could explain the higher OS found.

Surgery is the gold standard treatment for early RCC, with ablative techniques reserved for patients not surgical candidates [16]. In this regard, SBRT emerges as a promising non-invasive alternative for them due to excellent LC rates and limited toxicity [16–18], which is important in view of the increasing diagnosis of RCC and AM in an ageing population with comorbidities [16, 19].

Minimally invasive techniques should be used in patients with comorbidities and when renal function must be preserved, as they have demonstrated some advantages related to hospitalization, comorbidity, and side effects [5, 20, 21]. Recently, the International Renal Radiation Oncology Radiosurgery Consortium (IROCK) reported that patients treated with single fraction SBRT have a lower risk of distant progression or death from cancer [18], which agrees with our results regarding better LC and OS in shorter treatments.

In larger AM, may be technically challenging to completely resect the lesion without causing associated morbidity (23.1%) [22]. For this reason, less invasive options are being increasingly explored. A recent meta-analysis including 959 patients reported LC rate at 1-year of 80% and a rate of grade 3 adverse event of 13.6% [2, 23] for percutaneous ablation. SBRT could provide a non-invasive ablative treatment, to treat larger tumors, and evidence suggests an excellent LC and low toxicity rates [4].

Franzese et al. confirm the efficacy and safety of SBRT in 149 AM. OS at one and two years was 72.3% and 53.5% respectively, median SBRT dose was 40 Gy. Significant independent factors in univariate analysis were (1) performance status correlated with survival (*p* = 0.006); (2) primary lung tumor, with 1- and 2-year LC of 85.4% and 79.2% (*p* = 0.021), and (3) BED10 (*p* = 0.036). PFS at one and two years was 37.7% and 24.8%. Median time to polymetastatic disease was 11.3 months. Grade 1 and 2 toxic effects occurred in 21 (14.7%) and 3 (2.1%) patients [2]. Our series is obviously much shorter, but we obtained a 2-year OS of 60%. Four clinical factors were significantly associated with exitus: prior chemotherapy (*p* < 0.05), number of SBRT fractions (*p* < 0.05,), prescribed dose (*p* < 0.01) and lesion volume (*p* < 0.01). Konig et al. recently published the results of 28 patients treated with a median BED10 of 75 Gy, with good tolerance and only grade 1 and 2 acute toxicity in 32% of patients [2, 24]. This is similar in our study with the 88% of patients received SBRT schedules with BED ≥ 100 Gy. In our series, only one patient showed grade 3 toxicity (duodenal ulcer). In addition, patients without renal toxicity had 8-session schedules as did 72.72% of patients without ureteral toxicity and had smaller lesions.

The prospective, randomized SABR-COMET trial has demonstrated increased PFS and OS in multiple primary histologies with SBRT [25]. SBRT is also effective and safe in oligometastatic RCC patients, increasing OS and delaying the use of second-line systemic therapy [26]. Cassamassima et al. with their retrospective series of 48 patients with adrenal metastases reported a one- and two-year OS of 39.7% and 14.5%, respectively, and a one-year disease control rate of 9%, because of distant failure [8, 27].

In metastatic disease, systemic treatment is a priority, but it must be considered that combined with local treatments presents synergistic effects [28]. This is consistent with our finding, which shows that patients treated with SBRT after chemotherapy have better LC and a 6-month disease-free interval. Recently, the NIVES study suggests that the combination of SBRT and Nivolumab is an excellent treatment in patients with oligometastatic or oligoprogressive disease [29].

In AM, SBRT performs similarly to surgery in terms of LC and has very few serious side effects (up to 1.8%), even less than local ablative techniques. For palliative RT, the LC for SBRT is 84.8% and for standard RT it is 44% [2, 25].

Surgery is the gold standard treatment for these pathologies and allows histological characterization of the lesion, nevertheless minimally invasive techniques are a therapeutic alternative for fragile patients.

Our main limitation was the limited and mixed sample. Nonetheless, the analytical strategy was adjusted to this sample size; however, the findings of this study should be interpreted cautiously. Other limitations are the retrospective nature of the study and not to have a control group.

Grouping RCC and AM in the same group seems controversial but from a radiotherapy point of view it makes sense because the dose constraints and possible side effects are the same since they are in the same anatomical area.

SBRT could be a non-invasive alternative for many patients with RCC and AM being considered medically inoperable.

In our series LC time was greater than 6 months in 87% of patients. Patients treated with higher hypofrationation got better LC. Our study agrees with the SAFRON-II clinical trial which demonstrates the value of single -fraction SBRT for lung oligometasteses (28 Gy x 1 vs. 12 Gy x 4) [30]. So, less fractions means more efficacy.

The 88% of patients received SBRT schedules with BED ≥ 100 Gy. The 73.68% of patients achieved an image complete response. Patients who received prior QT or surgery had better complete response and disease-free survival (> 6 months) respectively, which may be due to a synergistic effect.

SBRT could be an alternative to surgery with high LC and OS for RCC and AM as initial treatment as well as for those lesions previously treated with surgery and/or chemotherapy.

## Electronic supplementary material

Below is the link to the electronic supplementary material.

Supplementary Material 1

Supplementary Material 2

Supplementary Material 3

## Data Availability

The datasets generated during and/or analyzed during the current study are available from the corresponding authors on reasonable request.
