# Supplementary material for: Stereotactic Body Radiotherapy: is less fractionation more effective in adrenal and renal malignant lesions?
Source: World J Urol. 2024 Jul 24;42(1):435. doi: 10.1007/s00345-024-05140-9 (PMC11269452; doi:10.1007/s00345-024-05140-9)
Supplement: Supplementary file 1 — Supplementary Material 1 [file 345_2024_5140_MOESM1_ESM.docx]

**Supplementary Table 1.** Devices, positioning systems and planning systems.

| **Device** | **Immobilization/Positioning system** | **Treatment Planning system (TPS)** |
| --- | --- | --- |
| Tomotherapy® | SIHO® diaphragmatic compressor “Breathing Dumping” (SIHO Medical Devices, Vigo, Pontevedra, Spain). | Pinnacle3 v9.10 Philips® (Philips Radiation Oncology Systems, Fitchburg, WI, USA) station and dosimetry in Tomotherapy Hy-Art Planning Station 5.1.1.6 Accuray ® (Accuray, Madison, WI, USA). |
| CyberKnife® | Common immobilization system for holding the position and 3 or 4 fiducials in the treatment area or close to it. | CyberKnife® software MultiPlan® version 4.6 (Accuray Incorporated, Sunnivale, CA, USA). |
| Elekta® LINAC | BodyFIX® Diaphragm Control from Elekta and a vacuum mattress. | Monaco version 5.00.04 (Elekta Instrument, AB, Stockholm, Sweden) for LINACs.  Pinnacle version 14.0 Philips® (Philips Radiation Oncology Systems, Fitchburg, WI, USA) for LINACs. |

Article title: Stereotactic Body Radiotherapy: Is less fractionation more effective in adrenal and renal malignant lesions?

Journal name: World Journal of Urology

Author names: Daniel Rivas, Alejandro de la Torre-Luque, Elena Moreno-Olmedo, Paloma Moreno, Vladimir Suárez, Ana Serradilla, Gregorio Arregui, David Álvarez, Morena Sallabanda, Antonio Lazo, María Isabel Núñez and Escarlata López.

Affiliation and e-mail address of the corresponding author: Department of Radiology and Physical Medicine, Granada University, Granada, Spain/Biopathology and Regenerative Medicine Institute (IBIMER), Centre for Biomedical Research, Granada University, Granada, Spain/Biosanitary Research Institute, ibs. Granada, Spain; isabeln@go.ugr.es
