# Supplementary material for: Stereotactic Body Radiotherapy: is less fractionation more effective in adrenal and renal malignant lesions?
Source: World J Urol. 2024 Jul 24;42(1):435. doi: 10.1007/s00345-024-05140-9 (PMC11269452; doi:10.1007/s00345-024-05140-9)
Supplement: Supplementary file 2 — Supplementary Material 2 [file 345_2024_5140_MOESM2_ESM.docx]

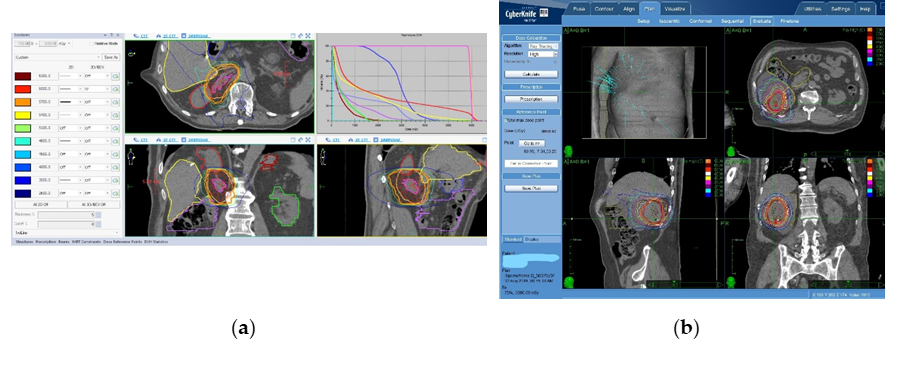


Supplementary Figure 1. LINAC and CyberKnife® dosimetry. a) Dosimetry in LINAC for AM and b) dosimetry in CyberKnife® for RCC.

Article title: Stereotactic Body Radiotherapy: Is less fractionation more effective in adrenal and renal malignant lesions?

Journal name: World Journal of Urology

Author names: Daniel Rivas, Alejandro de la Torre-Luque, Elena Moreno-Olmedo, Paloma Moreno, Vladimir Suárez, Ana Serradilla, Gregorio Arregui, David Álvarez, Morena Sallabanda, Antonio Lazo, María Isabel Núñez and Escarlata López.

Affiliation and e-mail address of the corresponding author: Department of Radiology and Physical Medicine, Granada University, Granada, Spain/Biopathology and Regenerative Medicine Institute (IBIMER), Centre for Biomedical Research, Granada University, Granada, Spain/Biosanitary Research Institute, ibs. Granada, Spain; isabeln@go.ugr.es
